# A novel and promising therapeutic approach for NSCLC: recombinant human arginase alone or combined with autophagy inhibitor

**DOI:** 10.1038/cddis.2017.137

**Published:** 2017-03-30

**Authors:** Weitao Shen, Xuyao Zhang, Xiang Fu, Jiajun Fan, Jingyun Luan, Zhonglian Cao, Ping Yang, Zhongyuan Xu, Dianwen Ju

**Affiliations:** 1 Department of Research Center for Clinical Pharmacology, Nanfang Hospital, Southern Medical University, Guangzhou 510515, China; 2 Department of Microbiological and Biochemical Pharmacy & The Key Laboratory of Smart Drug Delivery, Ministry of Education, School of Pharmacy, Fudan University, Shanghai 201203, China; 3 Department of Instrumental Analysis Center, School of Pharmacy, Fudan University, Shanghai 201203, China

## Abstract

Recombinant human arginase (rhArg), an enzyme capable of depleting arginine, has been shown to be an effective therapeutic approach for various cancers. Non-small-cell lung cancer (NSCLC), a histological subtype of pulmonary carcinoma, has a high rate of morbidity and mortality in the world. Thus, the need for novel and more effective treatment is urgent. In this study, it is the first time to report that rhArg could induce significant cytotoxicity and caspase-dependent apoptosis in NSCLC cells. Subsequently, our research revealed that rhArg dramatically stimulated autophagic response in NSCLC cells, which was proved by the formation and accumulation of autophagosomes and the conversion of microtubule-associated protein light chain 3 (LC3) from LC3-I to LC3-II. Furthermore, blocking autophagy by chloroquine or LY294002 remarkably enhanced rhArg-induced cytotoxicity and caspase-dependent apoptosis, suggesting that autophagy acted a cytoprotective role in rhArg-treated NSCLC cells. Further experiments showed that two signaling pathways including the Akt/mTOR and extracellular signal-regulated kinase pathway, and mitochondrial-derived reactive oxygen species (ROS) production were involved in rhArg-induced autophagy and apoptosis. Meanwhile, N-acetyl-L-cysteine, a common antioxidant, was employed to scavenge ROS, and we detected that it could significantly block rhArg-induced autophagy and cytotoxicity, indicating that ROS played a vital role in arginine degradation therapy. Besides, xenograft experiment showed that combination with autophagy inhibitor potentiated the anti-tumor efficacy of rhArg *in vivo*. Therefore, these results provided a novel prospect and viewpoint that autophagy acted a cytoprotective role in rhArg-treated NSCLC cells, and treatment with rhArg alone or combined with autophagy inhibitor could be a novel and promising therapeutic approach for NSCLC *in vivo* and *in vitro*.

Lung carcinoma is the main cause of mortality in males among all types of cancers. Non-small-cell lung cancer (NSCLC), one histology subtype of lung cancer, accounts for more than 80% of all lung cancer patients.^1, 2^ Depending on the staging of lung cancer, patients might be eligible for current standard therapies ranging from surgery to radiation to chemotherapy as well as targeted therapy.^3^ However, with the high recurrence rate and poor prognosis of NSCLC, current treatments only provide limited improvement in overall survival, thus developing new therapy strategies is urgently needed for NSCLC treatment.^4^

Arginine, a semi-essential amino acid, plays a significant role in some metabolic processes including protein synthesis, ornithine cycle and biosynthesis of creatine, polyamine and other important substances for survival.^5, 6^ Recently, arginine depletion has been shown to be considerably effective anti-cancer therapy *in vitro*.^7, 8^ Further researches have suggested that weakness or deficiency in either argininosuccinate synthetase (ASS) or ornithine transcarbamylase (OTC) expression contributed to arginine auxotrophy such as triple-negative breast cancers and laryngeal squamous cell carcinoma, which rendered them susceptible to arginine deprivation.^9, 10^ As arginase involves in the ornithine cycle, and serves as a catalyst converting arginine to ornithine and urea, recombinant human arginase (rhArg) has been being studied and developed for anti-tumor therapy and demonstrated to have a therapeutic effect in some tumors.^11^ However, both the efficiency and the potential mechanism of rhArg-based therapy for NSCLC have not yet been well evaluated.

Autophagy, a cellular response to external stressors and internal needs, serves as an evolutionarily conserved metabolic process responsible for the degradation of bulk superfluous, dysfunctional proteins and organelles.^9^ It has been widely reported that depleting amino acids could stimulate autophagy, in response to nutrition stress.^12^ Mounts of studies indicated that autophagy played a cytoprotective role in pathogenesis process and antitumor therapy.^13^ Notably, more and more evidences indicated that reactive oxygen species (ROS) were also strongly involved in the response to cancer therapeutics.^14^ Thus, stimulation of autophagy by rhArg through depleting arginine and mitochondrial-derived ROS production have been highlighted in the treatment with rhArg for NSCLC.

In our study, we explored the potential effect of rhArg on NSCLC cells, and it was the first report that rhArg showed remarkable cytotoxicity and induced caspase-dependent apoptosis in NSCLC cells. Meanwhile, we found that autophagy was activated by rhArg, inducing the activity of LC3, which was converted from LC3-I to LC3-II, while both the Akt/mTOR and extracellular signal-regulated kinase (Erk) signaling pathways were involved in autophagic process. Furthermore, inhibiting rhArg-induced autophagy by pharmacological inhibitors chloroquine (CQ) or LY294002 significantly enhanced rhArg-induced cytotoxicity, revealing autophagy played a cytoprotective role in rhArg-based therapy of NSCLC. In addition, we found that ROS was also involved in rhArg-induced autophagy and cytotoxicity. Therefore, setting autophagy as the breakthrough point, rhArg could potentially be a novel and promising approach for NSCLC treatment.

## Results

### RhArg induced potent cytotoxicity in NSCLC cells

**Figure 1:**
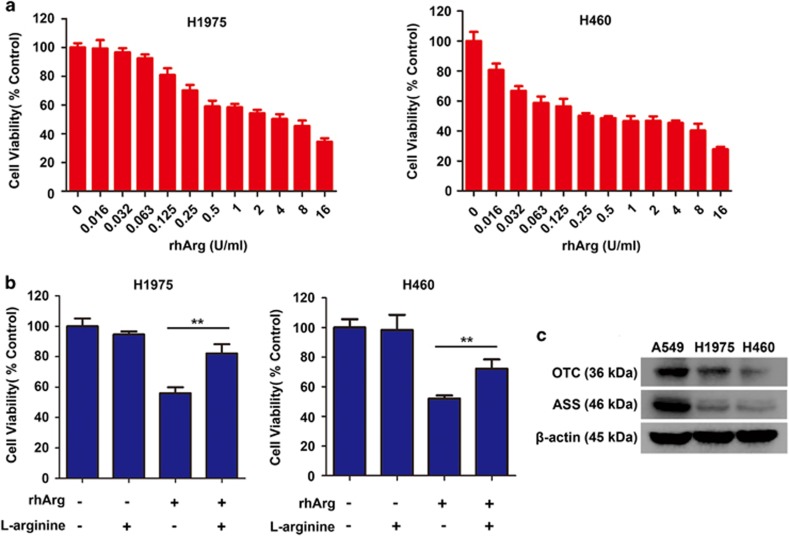
RhArg induced potent arginine-degrading-related cytotoxicity in NSCLC cells. (**a**) H1975 and H460 cells were incubated for 72 h in the presence or absence of different concentrations of rhArg. (**b**) H1975 and H460 cells were incubated with 2 U/ml of rhArg, respectively, in the presence or absence of 8 mM arginine for 72 h. Results were expressed as mean±S.D. and analyzed by Student's *t*-test (two-tailed). **P<0.01 (**c**) The protein expression of ASS and OTC was measured by western blot analysis in A549, H1975 and H460 cells. A549 cells were used as positive control

Firstly, we investigated whether rhArg could cause cytotoxicity in NSCLC cell *in vitro*. Two NSCLC cells were incubated with increasing concentrations of rhArg for 72 h. As exhibited in Figure 1a, the cytotoxicity was significantly induced by rhArg in a dose-dependent manner in H1975 and H460 cells.

Furthermore, when H1975 and H460 cells were treated with rhArg and supplemented with exogenous L-arginine at the same time, the cytotoxicity induced by rhArg could be partially rescued (Figure 1b). In order to further determine the relationship between cells' sensitivity to arginase treatment and arginine, we evaluated ASS and OTC, key enzymes of arginine synthesis, by western blot assay (Figure 1c, Supplementary Figures 1a and b). A549 cells were employed as positive control for being evidenced to have resistance to rhArg and high expression level of ASS and OTC proteins.^15, 16^ And, we found that the expression of ASS and OTC proteins in two NSCLC cells was weak, which was consistent with the sensitivity to rhArg.

Collectively, these data revealed that rhArg could induce potent cytotoxicity in NSCLC cells.

### RhArg induced caspase-dependent apoptosis in H1975 cells

**Figure 2:**
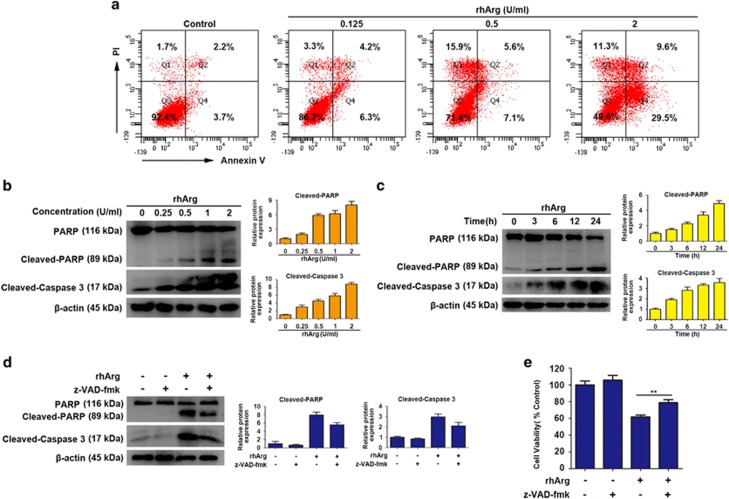
RhArg induced caspase-dependent apoptosis in H1975 cells. (**a**) After treatment with different concentrations of rhArg for 72 h, H1975 cells were stained with Annexin V/PI and analyzed by flow cytometry. (**b**) H1975 cells were dose dependently treated with rhArg for 24 h. (**c**) H1975 cells were time dependently treated with 2 U/ml of rhArg. (**d**) H1975 cells were incubated with 2 U/ml of rhArg in the presence or absence of 20 *μ*M z-VAD-fmk for 24 h. (**b–d**) Western blot analysis was performed to assess the expression level of PARP, cleaved-PARP and cleaved-caspase 3. Densitometric values were quantified using the ImageJ software and normalized to control. The values of control were set to 1. The data are presented as means±S.D. of three independent experiments. (**e**) H1975 cells were incubated with 2 U/ml of rhArg in the presence or absence of 20 *μ*M z-VAD-fmk for 48 h. Results were expressed as mean±S.D. and analyzed by Student's *t*-test (two-tailed). **P<0.01

For many antitumor drugs-induced cell death through apoptosis, we used flow cytometry to determine whether apoptosis was triggered in rhArg-treated cells. As performed in Figure 2a, rhArg induced remarkable apoptosis with the increase of drug concentrations for 72 h. What's more, the expression level of cleaved PARP and cleaved caspase 3 increased in a dose- and time-dependent manner in western blot analysis, further demonstrated that rhArg induced caspase-dependent apoptosis (Figures 2b and c).

Then we used benzyloxycarbonyl Val-Ala-Asp (O-methyl)-fluoro-methylketone (z-VAD-fmk), an irreversible general caspase-family inhibitor, to further demonstrate the caspase-dependent apoptosis. When H1975 cells were pre-treated with z-VAD-fmk, the expression of cleaved caspase 3 and cleaved-PARP significantly decreased (Figure 2d). In addition, cytotoxicity induced by rhArg could be partially rescued by z-VAD-fmk (Figure 2e).

Taken together, these results suggested that rhArg induced caspase-dependent apoptosis in H1975 cells.

### RhArg induced autophagy in H1975 cells

**Figure 3:**
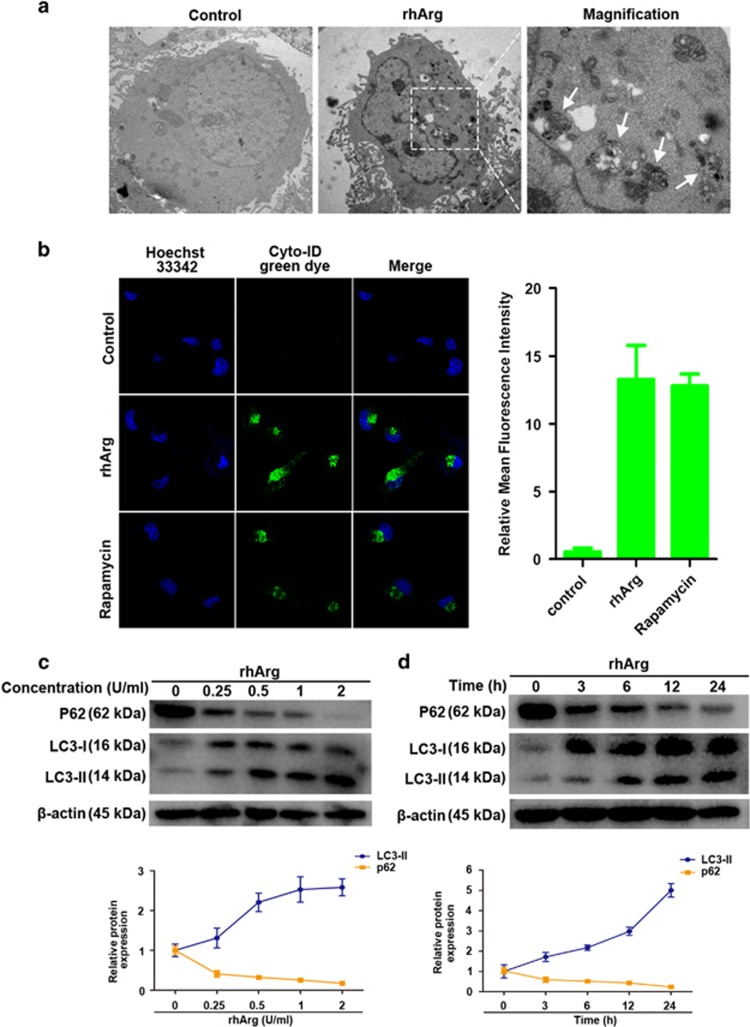
Autophagy was induced by rhArg in H1975 cells. (**a**) H1975 cells were incubated with or without 2 U/ml of rhArg for 24 h and TEM was employed to detect the autophagosomes, and the magnified view of the electron photomicrograph exhibited autophagosomes. The left and middle micrographs were taken at × 5000, whereas the right was taken at × 15 000. (**b**) After stained with Cyto-ID Green dye, the formation of autophagic vesicles in H1975 cells treated with 2 U/ml of rhArg for 24 h was found through laser confocal microscopy. The autophagy inducer rapamycin (50 nM) was chosen as a positive control. Green (Cyto-ID) dots in cells were counted by the ImageJ software (*n*=3, means±S.D.). Confocal micrographs were taken at × 40. (**c**) H1975 cells were incubated with different concentrations of rhArg for 24 h, then autophagy associate protein LC3-I/II and p62 was detected by western blot analysis. Densitometric values were quantified using the ImageJ software and normalized to control. The values of control were set to 1. The data are presented as means±S.D. of three independent experiments. (**d**) H1975 cells were incubated with 2 U/ml of rhArg for different time, then autophagy associate protein LC3-I/II and p62 were detected by western blot analysis. Densitometric values were quantified as described in **c**

When cells suffer from some environmental stresses such as nutritional starvation including arginine depletion, autophagy, an adaptive cellular process for survival, occurs to maintain nutrient homeostasis by degrading damaged organelles and protein aggregates.^17, 18, 19^ To investigate whether rhArg could induce autophagy in NSCLC cells, we used three persuasive approaches of transmission electron microscopy (TEM), laser confocal microscopy, as well as western blot assay in this study. First, after H1975 cells were treated with rhArg for 24 h, large amounts of intracellular autophagosomes could be detected through TEM compared with control (Figure 3a). Second, by staining of H1975 cells with Cyto-ID Green dye, the formation of autophagic vesicles was detected in rhArg-treated cells through laser confocal microscopy (Figure 3b). Autophagy inducer rapamycin was chosen as a positive control. The emergence of punctuate fluorescent dots revealed that rhArg dramatically stimulated autophagy in H1975 cells. Finally, we examined the expression of autophagy marker LC3-II and p62. Consistent with above, the high expression of LC3-II in company with the transformation of LC3-I to LC3-II was found in cells after treatment with rhArg in series concentrations and time (Figures 3c and d). While the p62 expression level decreased in time- and concentration-dependent way in rhArg-treated H1975 cells (Figures 3c and d).

All these results forcefully demonstrated that rhArg induced autophagy in H1975 cells.

### Autophagic flux was detected in rhArg-treated H1975 cells

**Figure 4:**
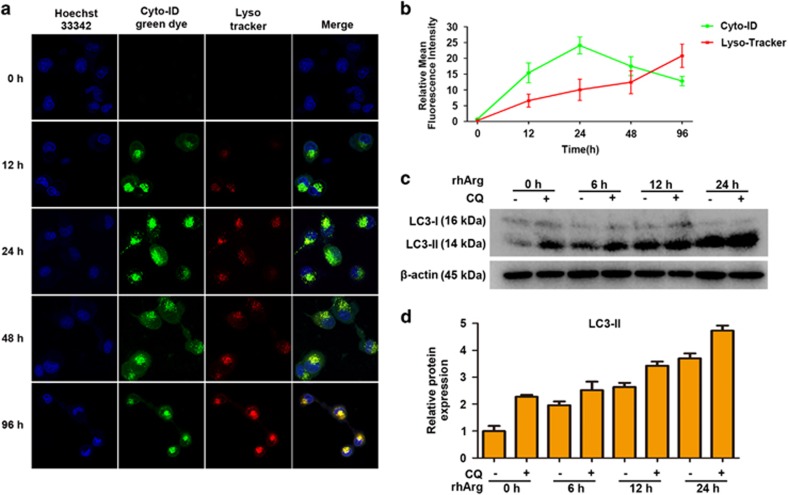
Autophagic flux was detected in H1975 cells after rhArg treatment. (**a**,**b**) H1975 cells were stained with Cyto-ID and LysoTracker Red after exposed to 2 U/ml of rhArg for the indicated times. Confocal micrographs were taken at × 40. Green (Cyto-ID) and red (LysoTracker Red) dots in cells were counted by the ImageJ software (*n*=3, means±S.D.). (**c**,**d**) H1975 cells were treated with 2 U/ml of rhArg for different time in the presence or absence of 20 *μ*M CQ. Cell lysates were analyzed by immunoblot analysis. Densitometric values were quantified using the ImageJ software and normalized to control. The values of control were set to 1. The data are presented as means±S.D. of three independent experiments

Autophagy is a complex process, which begins with nucleation, followed by autophagosomes formation, fusion of autophagosomes to lysosomes and degradation of cellular contents in lysosomes.^20^ In order to further investigate rhArg-stimulated autophagic activity, autophagic flux was evaluated in rhArg-treated H1975 cells. In our study, laser confocal microscopy was applied to examine autophagic flux. After cells were treated with rhArg for different time, we stained H1975 cells by Cyto-ID and LysoTracker. Notably, the formation of autophagosomes dyed with green fluorescence was detected within 12 h, then accumulated to the extreme within 24 h, finally it decreased in green dots. On the other hand, red fluorescence always increased during the experiment. In merge images, the yellow fluorescence occurred at 48 and 96 h, revealing that autophagosomes had been transforming into autolysosomes gradually (Figures 4a and b). In parallel, H1975 cells were treated with rhArg and/or CQ, a lysosome pH neutralizing agent, for different time. Then, the results showed that additional treatment with CQ could improve the expression of LC3-II in H1975 cells compared with incubating rhArg alone for increasing time (Figures 4c and d).

All these data demonstrated that rhArg could trigger autophagic flux in H1975 cells.

### Blocking autophagy enhanced cytotoxicity and apoptosis induced by rhArg in H1975 cells

**Figure 5:**
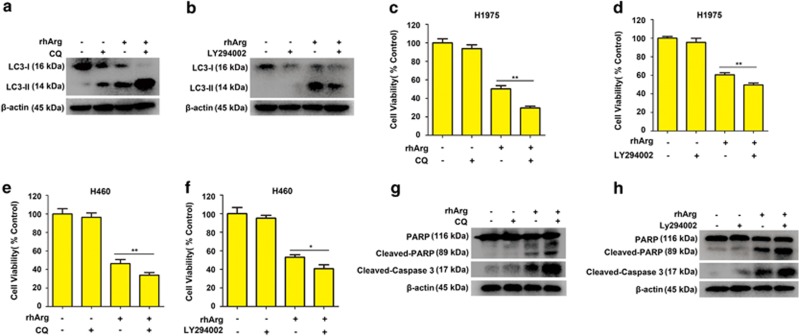
Suppressing autophagy promoted cytotoxicity and caspase-dependent apoptosis induced by rhArg in NSCLC cells. (**a**,**b**) H1975 cells were incubated with 1 U/ml of rhArg for 24 h in the presence or absence of 20 *μ*M CQ or 15 *μ*M LY294002, and the expression of LC3-I/II was measured by western blot analysis. (**c**) H1975 cells were incubated with 1 U/ml of rhArg for 96 h in the presence or absence of 20 *μ*M CQ. (**d**) H1975 cells were incubated with 1 U/ml of rhArg for 72 h in the presence or absence of 15 *μ*M LY294002. (**e**,**f**) H460 cells were incubated with 2 U/ml of rhArg for 72 h in the presence or absence of 20 *μ*M CQ or 15 *μ*M LY294002. (**c–f**) The cell viability was determined by MTT assay. Results were expressed as mean±S.D. and analyzed by Student's *t*-test (two-tailed). *P<0.05, ** *P*<0.01. (**g** and **h**) H1975 cells were incubated with 1 U/ml of rhArg for 24 h in the presence or absence of 20 *μ*M CQ or 15 *μ*M LY294002, and the expression of PARP, cleaved-PARP and cleaved-caspase 3 was measured by western blot analysis

Recently, some researches have confirmed the cytoprotective effect of autophagy and presented a novel idea that inhibition of autophagy could strengthen the efficacy of anti-tumor drugs in cancer therapy.^21, 22, 23^ In this research, we explored whether blockage of rhArg-induced autophagy could enhance rhArg-induced cytotoxicity in H1975 cells. CQ and LY294002, a lysosome inhibitor and a phosphatidylinositol 3-kinases inhibitor, respectively,^24^ were applied to inhibit rhArg-induced autophagy. As exhibited in Figures 5a, b, Supplementary Figures 2a and b, autophagy was significantly suppressed when cells were co-treated with rhArg and CQ or LY294002, respectively. Furthermore, as shown in Figures 5c and d, combination with rhArg and CQ or LY294002 resulted in more significant cytotoxicity in H1975 cells. Similarly, we also detected that CQ and LY294002 could enhance rhArg-induced cytotoxicity in H460 cells (Figures 5e and f). In addition, after suppressing rhArg-induced autophagy by CQ or LY294002, caspase-dependent apoptosis was remarkably encouraged in H1975 cells (Figures 5g, h and Supplementary Figures 2c–f).

Taken together, our data indicated that inhibiting autophagy enhanced rhArg-induced cytotoxicity and caspase-dependent apoptosis in H1975 cells.

### Akt/mTOR and Erk molecular signaling pathways acted an important role in rhArg-induced autophagy in H1975 cells

**Figure 6:**
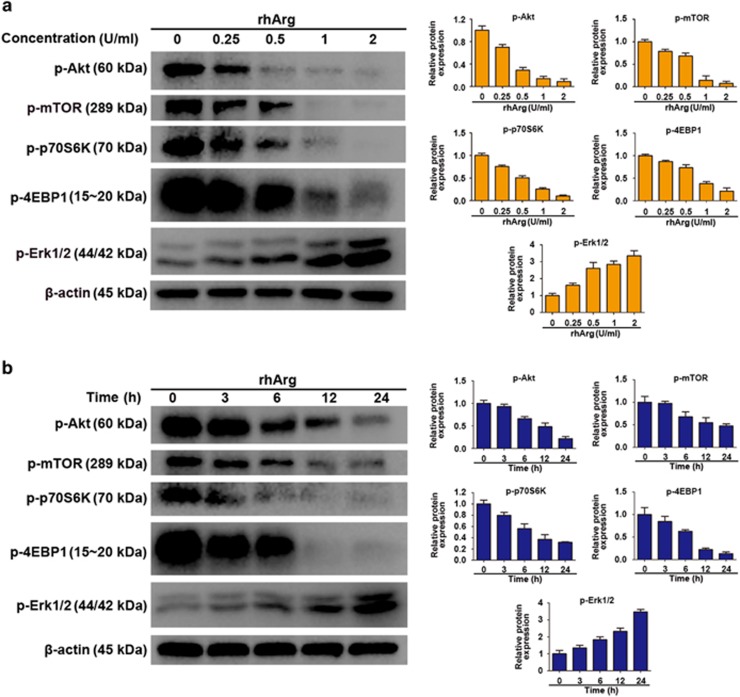
Akt/mTOR and Erk signaling pathway played a critical role in rhArg-induced autophagy in H1975 cells. (**a**) H1975 cells were incubated with different concentrations of rhArg for 24 h, then western blot was performed to analyze the protein p-Akt, p-mTOR, p-P70S6K, p-4EBP1 and p-Erk 1/2. Densitometric values were quantified using the ImageJ software and normalized to control. The values of control were set to 1. The data are presented as means±S.D. of three independent experiments. (**b**) H1975 cells were incubated with 2 U/ml of rhArg for indicated time, then western blot was performed to analyze the protein p-Akt, p-mTOR, p-P70S6K, p-4EBP1 and p-Erk 1/2. Densitometric values were quantified as described in **a**

Akt/mTOR is a crucial signaling pathway responsible for cell differentiation, proliferation, survival, metabolism, apoptosis and motility regulation, and it acts as a crucial role in regulating autophagy in eukaryotic cells.^25, 26^ In addition, Akt/mTOR activation phosphorylates protein S6 kinase (p70S6K) and 4E-binding protein 1 (4EBP1), which leads to protein synthesizing and cell growth. When cells suffer nutrient depletion, some cellular survival process such as autophagy can be activated through inhibiting Akt/mTOR signaling pathway.^27^ To investigate whether Akt/mTOR pathway participated in autophagic response induced by rhArg, we explored molecular signaling pathways of rhArg-induced autophagy. As shown in Figures 6a and b, rhArg significantly decreased the phosphorylation of Akt in a concentration- and time-dependent way. The expression level of phosphorylation of mTOR, an important downstream regulator, was significantly reduced. Moreover, both 4E-BP1 and p70S6K, the downstream components of Akt/mTOR pathway, also performed remarkably decreased phosphorylation.

There are some studies, which had demonstrated that Erk takes part in the regulation of autophagy in cells that suffer amino-acid deprivation.^28^ In our research, the phosphorylation of Erk was examined through western blot assay, and was observed increasing in a concentration- and time-dependent manner in rhArg-treated H1975 cells (Figures 6a and b).

Collectively, all these results demonstrated the crucial role of Akt/mTOR and Erk signaling pathways in rhArg-induced autophagy in H1975 cells.

### Mitochondrial-derived ROS production was involved in rhArg-induced autophagy and cytotoxicity in H1975 cells

**Figure 7:**
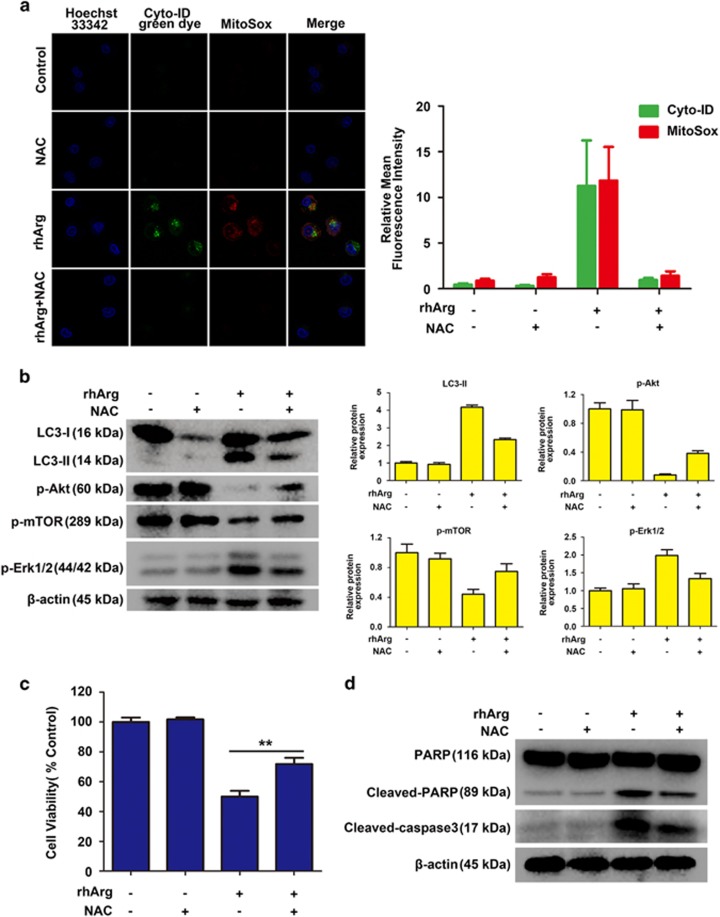
Mitochondrial-derived ROS production was involved in rhArg-induced autophagy and cytotoxicity in H1975 cells. (**a**) H1975 cells were treated with 2 U/ml of rhArg in the presence or absence of 5 mM NAC for 72 h and stained by MitoSox and Cyto-ID green dye to detect the ROS and autophagy. Confocal micrographs were taken at × 40. Green (Cyto-ID) and red (MitoSox) dots in cells were counted by the ImageJ software (*n*=3, means±S.D.). (**b**) H1975 cells were incubated with 2 U/ml of rhArg for 24 h in the presence or absence of 5 mM NAC and the expression of LC3-I/II, p-Akt, p-mTOR and p-Erk 1/2 was measured by western blot analysis. Densitometric values were quantified using the ImageJ software and normalized to control. The values of control were set to 1. The data are presented as means±S.D. of three independent experiments. (**c**) H1975 cells were treated with 2 U/ml of rhArg in the presence or absence of 5 mM NAC for 72 h, and cell viability was analyzed by MTT assay. Results were expressed as mean±S.D. and analyzed by Student's *t*-test (two-tailed). **P<0.01 (**d**) H1975 cells were incubated with 2 U/ml of rhArg for 24 h in the presence or absence of 5 mM NAC and the expression of PARP, cleaved-PARP and cleaved-caspase 3 was measured by western blot analysis

Amounts of evidence indicated that ROS is involved in autophagy stimulated by antitumor drugs for cancer therapy.^29^ To assess the occurrence of ROS in H1975 cells after treatment with rhArg, we applied Cyto-ID Green dye and MitoSox red dye to stain cells incubated with rhArg and/or a common antioxidant of N-acetyl-L-cysteine (NAC) for 72 h. As exhibited in Figure 7a, the formation of ROS and autophagy was detected by the signal of red and green fluorescence in cells treated with rhArg alone. However, in H1975 cells that were treated with NAC and rhArg, both autophagy and ROS significantly decreased, further suggesting that rhArg-stimulated autophagy was attenuated by NAC through inhibiting ROS. Meanwhile, we observed that the conversion of LC3-I to LC3-II could be notably attenuated after pretreatment with NAC, indicating that the antioxidant exhibited an inhibiting effect on rhArg-triggered autophagy (Figure 7b). Furthermore, after pretreatment with NAC, the decreased expression of phosphorylation of Akt and mTOR induced by rhArg was significantly reversed, and the phosphorylation of Erk was also remarkably decreased, which might reveal the underlying relationship between ROS and the molecular signaling pathway of Akt/mTOR and Erk pathway. Interestingly, NAC could partially rescue the cytotoxicity and apoptosis in H1975 cells after rhArg treatment (Figures 7c, d, Supplementary Figures 3a and b).

Thus, these data indicated that mitochondrial-derived ROS production was involved in rhArg-triggered autophagy and cytotoxicity in H1975 cells.

### Inhibition of autophagy potentiated the anti-tumor efficacy of rhArg *in vivo*

**Figure 8:**
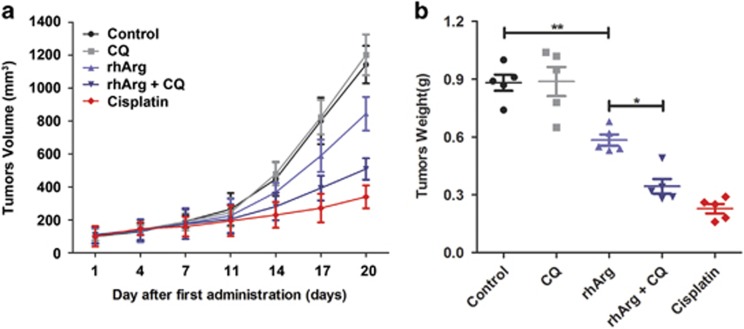
rhArg alone or combined with autophagy inhibitor CQ exhibited remarkable anti-tumor effect *in vivo*. (**a**) Tumor growth between different groups was measured and calculated by an ellipsoid volume formula (length × width^2^/2) twice a week. (**b**) The tumor weight of different groups was measured after 20-day treatment (*P<0.05, **P<0.01)

To evaluate the anti-tumor efficacy of rhArg alone or in combination with autophagy inhibitor *in vivo*, NSCLC xenograft models were also established subcutaneously in nude mice. After the tumor-bearing mice were randomized into five groups (five mice in each group), isotonic saline, CQ, rhArg and cisplatin were intraperitoneally injected. After 20-day treatment, the average values of tumor weight of saline, CQ, rhArg, rhArg in combination with CQ, cisplatin were 882, 888, 584, 344 and 228 mg. We found that rhArg could inhibit the subcutaneous tumor growth as the potent growth inhibition *in vitro*. Furthermore, when NSCLC-bearing mice were treated with rhArg in combination with autophagy inhibitor CQ, the anti-tumor efficacy was significantly enhanced (Figure 8). In addition, no apparent body weight loss was observed during the treatment (Supplementary Figure 4), which indicated that our designed treatments might be safe *in vivo*.

Thus, rhArg exhibited remarkable anti-tumor effect *in vivo*, suppression of autophagy by CQ potentiated the anti-tumor efficacy of rhArg *in vivo*.

## Discussion

Despite the improvement in clinical outcomes such as new chemotherapeutics or molecular targeted therapies for anaplastic lymphoma kinase or epidermal growth factor receptor, the overall survival of NSCLC still remains no significant improvements.^2^ So, it is urgent to develop a novel and more effective therapeutic approach to solve the dilemma.

As there exist significant differences in metabolism between cancer cells and normal cells, it may be a breakthrough to discover new anti-tumor strategies.^30^ As reported in recent studies, most carcinoma cells characterized weakness or deficiencies of ASS or OTC,^9^ contrary to normal cells. Thus, arginine depletion could be a potential therapeutic approach for malignant tumors, and the arginine biosynthesis key enzyme OTC and ASS were considered as effective and sensitive indicators in the arginine depletion therapy.

Recently, rhArg is being evaluated in phase II trials for hepatic cellular cancer.^31^ Meanwhile, rhArg has been demonstrated to be significant cytotoxicity in triple-negative breast cancers and laryngeal squamous cell carcinoma.^9, 10^ However, it has never been reported whether NSCLC cells were sensitive to rhArg treatment.

In this study, it was the first report that rhArg had cytotoxicity effect on NSCLC cells, which could be rescued by exogenous arginine, proving that rhArg functioned through arginine depletion. As shown above, ASS and OTC, the synthesizing arginine key enzymes, were weakly expressed in NSCLC H1975 and H460 cells, resulting in their sensitivity to rhArg. Consequently, NSCLC H1975 cells were chosen as the model cells for following experiments. In our research, we detected that rhArg induced apoptosis in NSCLC H1975 cells, demonstrated to be caspase-dependent. Then, through a series of standard methods, including TEM, laser confocal microscopy as well as western blot assay, the activities of autophagosomes and LC3 proteins were observed, which strongly confirmed the occurrence of autophagy in rhArg-based treatment for H1975 cells. In addition, we also found autophagic flux that vividly indicated the process of autophagy in rhArg-treated H1975 cells.

However, what role does autophagy play in cell death? Although autophagy has been discovered for more than 50 years, it is still controversial to define the character of autophagy in cell growth and death.^32^ Therefore, there are three aspects of this problem need to be addressed. First, more recent studies reveal that autophagy may act primarily as a cytoprotective role to maintain energy homeostasis and nutritional requirements during starvation conditions. Consequently, the second aspect involves the cytoprotective mechanism. In tumor cells, autophagy might contribute to chemotherapy resistance, causing undesired survival of tumor cells. Thus, regulating autophagy according to disease contexts, and balancing the advantages and disadvantages of autophagy stimulation or inhibition are future goals. Third, a deeper understanding of the regulatory mechanism of autophagy was urgently needed, and the underlying molecule mechanism of autophagy should be further explored.

To investigate the function of autophagy in rhArg-induced cytotoxicity and caspase-dependent apoptosis of H1975 cells, CQ and LY294002, two common autophagy inhibitors, were employed in our study. Combined with CQ or LY294002, the cytotoxicity and caspase-dependent apoptosis triggered by rhArg could be significantly enhanced, indicating autophagy played a cytoprotective function in rhArg-treated H1975 cells. Thus, inhibiting autophagy could become a promising approach to enhance the anti-NSCLC efficacy of rhArg. Finally, we investigated the underlying mechanism of rhArg-triggered autophagy in H1975 cells. As previous study shown, two signaling pathways of Akt/mTOR and Erk signaling pathway acted an important role in arginine depletion therapy for some tumors such as non-Hodgkin's lymphoma cells.^33^ In our research, after treatment with rhArg we detected that the expression of phosphorylation of Akt and mTOR was attenuated in a time- and dose-dependent manner in H1975 cells. Meanwhile, 4EBP1 and p70S6K, two downstream components of Akt/mTOR pathway, also performed remarkably decreased phosphorylation, whereas the phosphorylation of Erk increased in Erk signaling pathway. In addition, the role of ROS was investigated, after treatment with rhArg, over-accumulation of ROS was observed in H1975 cells. Furthermore, suppression of ROS by NAC could significantly weaken rhArg-induced autophagy and cytotoxicity. All these results indicated that both two signaling pathways of Akt/mTOR and Erk pathway, and ROS took part in autophagy triggered by rhArg in H1975 cells. Thereafter, we investigated the antitumor efficacy of rhArg *in vivo*, and it was detected that the growth of NSCLC xenograft tumors could be inhibited by rhArg. Moreover, combination with autophagy inhibitor significantly enhanced the antitumor efficacy of arginine deprivation *in vivo*.

Several groups have studied the possible mechanism of arginine deprivation-induced cytotoxicity and tried to reveal the role of autophagy in the cell death.^34, 35^ It was reported that pegylated arginine deiminase (ADI-PEG20) could induce caspase-dependent apoptosis in ASS1-lacking malignant lymphoid cells.^36^ However, in ASS-deficient glioblastoma cells and prostate cancer cells, ADI-PEG20-induced cytotoxicity was caspase-independent.^37, 38^ Combination with autophagy inhibitor potentiated the anti-tumor efficacy of ADI-PEG20 in these malignant tumors, indicating that autophagy played a cytoprotective role in arginine deprivation-induced cytotoxicity. Meanwhile, Qiu *et al.*^12^ reported that depriving arginine by ADI-PEG20 induced autophagy-dependent cytotoxicity in ASS1-deficient breast cancer cells. In this study, we reported for the first time that rhArg could induce remarkable caspase-dependent apoptosis in NSCLC cells and cytoprotective autophagy was also activated. Inhibiting rhArg-induced autophagy by pharmacological inhibitors significantly enhanced the anti-NSCLC efficacy *in vitro* and *in vivo*. To reveal the mechanism of mTOR complex 1 (mTORC1) in arginine deprivation-based therapy, Wang *et al.*^39^ showed that SLC38A9, an amino-acid transporter, signaled arginine sufficiency to mTORC1 and promoted mTORC1-mediated protein synthesis, indicating that blocking this signal could enhance the anti-tumor efficacy of amino-acid depletion enzyme. In our manuscript, our data also showed that inactivation of Akt/mTOR signaling pathways was involved in rhArg-induced autophagy in H1975 cells. Therefore, based on these data, researchers could explore more effective strategy to enhance the anti-tumor efficacy of arginine deprivation *in vivo* in the future study.

Compared with traditional cancer treatments, arginine deprivation therapy was characterized by its high efficiency, high specificity and low toxicity.^40^ In the era of precision medicine and immunotherapy for NSCLC, arginine deprivation can be employed in the multidisciplinary therapy for NSCLC, such as combination with traditional chemotherapy drugs or immunotherapy agents, which will further improve the therapeutic efficacy and reduce the side effects of medication and financial burden. In addition, the protein expression level of arginine synthesis enzymes in the urea cycle determined the sensitivity of NSCLC to rhArg-treatment, thus precise detection of arginine synthesis enzymes-related gene and mRNA can assess the efficacy of rhArg treatment.

In summary, through deprivation of arginine, rhArg had anti-tumor effect via inducing cytotoxicity and caspase-dependent apoptosis in the therapy for NSCLC H1975 cells, and the cytoprotective autophagy and autophagic flux were induced by rhArg. Then, blocking autophagy by autophagy inhibitors, such as CQ or LY294002, could significantly reinforce rhArg-induced cytotoxicity and caspase-dependent apoptosis in H1975 cells. Moreover, our study revealed that the potential mechanism of rhArg-induced autophagy was involved in both two signaling pathways of Akt/mTOR and Erk pathways, and mitochondrial-derived ROS production. Besides, *in vivo* study showed that combination with autophagy inhibitor potentiated the anti-tumor efficacy of rhArg *in vivo*. Therefore, we provided new insights that depriving arginine alone or in combination with blocking autophagy could be a novel and promising therapeutic approach for NSCLC.

## Materials and Methods

### Cell lines

NSCLC cells H1975 and H460 were both purchased from Cell Bank of the Chinese Academy of Sciences, Shanghai Branch (Shanghai, China). H1975 and H460 cells were cultured in 1640 medium, which is supplemented with 10% fetal bovine serum. About 100 U penicillin and 100 *μ*g streptomycin were added in the medium per milliliter. All cells were maintained at 37°C in a 5% CO_2_ incubator.

### Preparation of rhArg

RhArg was expressed and purified as described previously in our lab.^15^ The specific activity of rhArg with >95% purity was 200 U/ml. One unit of rhArg converted 1 *μ*M of arginine to 1 *μ*M of ornithine per minute under the assay conditions.

### Reagents

Cyto-ID Green dye and MitoSox were purchased from Enzo Life Sciences, Inc. (Farmingdale, NY, USA). LysoTracker was purchased from Invitrogen (SanDiego, CA, USA). FITC-AnnexinV/PI kit was purchased from BD Biosciences (Franklin Lakes, NJ, USA). The mouse antibody to ASS was obtained from BD Biosciences. All the other primary antibodies including antibodies to *β*-actin, LC3B, cleaved caspase 3, PARP, anti-cleaved PARP, phospho-mTOR (Ser2448), phospho-Akt (Ser473), p70S6 kinase phospho (pS371), phospho-4EBP1-pT45 were obtained from Cell Signaling Technology (Danvers, MA, USA). The secondary antibodies were purchased from MR Biotech (Shanghai, China). The autophagy inhibitors of the PI3K inhibitor LY294002 and the lysosomal inhibitor CQ, 3-(4,5-dimetrylthiazol-2-yl)-2, 5-diphenyltetrazolium bromide (MTT) as well as L-arginine were purchased from Sigma-Aldrich (St. Louis, MO, USA). Z-VAD-fmk and NAC were obtained from Beyotime Institute of Biotechnology (Haimen, Jiangsu Province, China).

### MTT analysis for cell viability assay

Cell viability was measured by MTT method. About 5 × 10^3^ cells per well were seeded in 96-well plates. After treatment with rhArg and/or autophagic inhibitors at indicated concentrations and time, 0.5 mg/ml of MTT was added to each well for 4 h at 37°C. Then dissolving formazan with DMSO, the optical density was measured by UV spectrophotometry at 570 nm.

### Apoptosis assay

Annexin V-FITC/PI Kit was employed for apoptosis analysis. After indicated treatment, cells were collected and washed by cold phosphate-buffered saline (PBS), then re-suspended in 1 × binding buffer at a concentration of 1 × 10^6^ cells/ml. The cells were stained with Annexin V-FITC/PI for about 15 min in the cell incubator. Cell apoptosis was analyzed by FACS Calibur flow cytometer (Becton-Dickinson, Fullerton, CA, USA).

### Immunoblot analysis

Cells were collected and washed with cold PBS, then kept at 0°C for about 30 min in Cell Lysis Buffer. After the lysates were centrifuged, the supernatants were transferred to fresh tubes. Measured by the bicinchoninic acid method, equal quantity of protein was separated by SDS-PAGE and subsequently transferred to PVDF membranes. The membranes were blocked with 3% bovine serum albumin powder in tris-buffered saline with Tween 20 (TBST) at room temperature overnight and then incubated with primary antibodies for 4 h at room temperature. After that, the membranes were washed three times each for at least 20 min, and incubated with secondary antibodies for 2 h at room temperature. At last, the signals were detected by enhanced chemiluminescence reagents. The ImageJ software (National Institutes of Health, Bethesda, MD, USA) was applied to quantify intensities in the resulting bands.

### Transmission electron microscopy

H1975 cells were treated with or without 2 U/ml of rhArg for 24 h, then collected and processed as reported in previous study.^41^ Samples were examined with a JEM 1400 transmission electron microscope (JEOL, Inc., Peabody, MA, USA) at 80 kV.

### Confocal microscopy

H1975 cells were treated with or without rhArg at indicated concentrations and time, then stained with Cyto-ID Green dye and/or LysoTracker Red, and/or MitoSOX at 37°C for 30 min. Cells treated with autophagy inducer rapamycin were employed as the positive control. All results were visualized by laser confocal microscopy (Carl Zeiss LSM710, Carl Zeiss, Oberkochen, Germany).

### *In vivo* study

The BALB/c nude mice used in this study were purchased from Shanghai Sippr-BK laboratory animal Co. Ltd and maintained under pathogen-free conditions in Fudan University. H1975 cells were harvested and suspended in culture medium and 1 × 10^7^ cells were subcutaneously injected to develop NSCLC xenograft model. As the tumors reached an average size of 100 mm^3^, the mice were randomly divided into five groups. Then rhArg (12 500 U/kg) and cisplatin (10 mg/kg) were administered intraperitoneally twice a week. CQ (50 mg/kg) and saline were administered intraperitoneally every day. Saline and cisplatin were used as negative and positive control, respectively. The size of tumor was calculated by an ellipsoid volume formula (length × width^2^/2) twice a week.

### Statistical analysis

The data conducted with GraphPad Prism 5 (GraphPad Software, Inc., La Jolla, CA, USA) were presented as means±S.D. through Student's *t*-test or one-way ANOVA. *P*-value <0.05 and *P*-value<0.01 were indicated with * and **, respectively, which were considered to be statistical significance of the differences between groups.
