# Supplementary material for: A novel and promising therapeutic approach for NSCLC: recombinant human arginase alone or combined with autophagy inhibitor
Source: Cell Death Dis. 2017 Mar 30;8(3):e2720–. doi: 10.1038/cddis.2017.137 (PMC5386540; doi:10.1038/cddis.2017.137)

**Supplementary Materials**

**A novel and promising** **therapeutic approach for NSCLC: recombinant human arginase alone or combined with autophagy inhibitor**

Weitao Shen^1,#^, Xuyao Zhang^2,#^, Xiang Fu^1^, Jiajun Fan^2^, Jingyun Luan^2^, Zhonglian Cao^3^, Ping Yang^3^, Zhongyuan Xu*^,1^, Dianwen Ju *^,2^

**Figure S1** **(a** and **b)**

**Figure S2** **(a**-**f)**

**Figure S3 (a** and **b)**

**Figure S4**

**Figure S1** **(a** and **b)** Statistics of the OTC and ASS expression in Figure 1c. Densitometric values were quantified using the ImageJ software and normalized to control. The values of control were set to 1. The data are presented as means ± SD of 3 independent experiments.


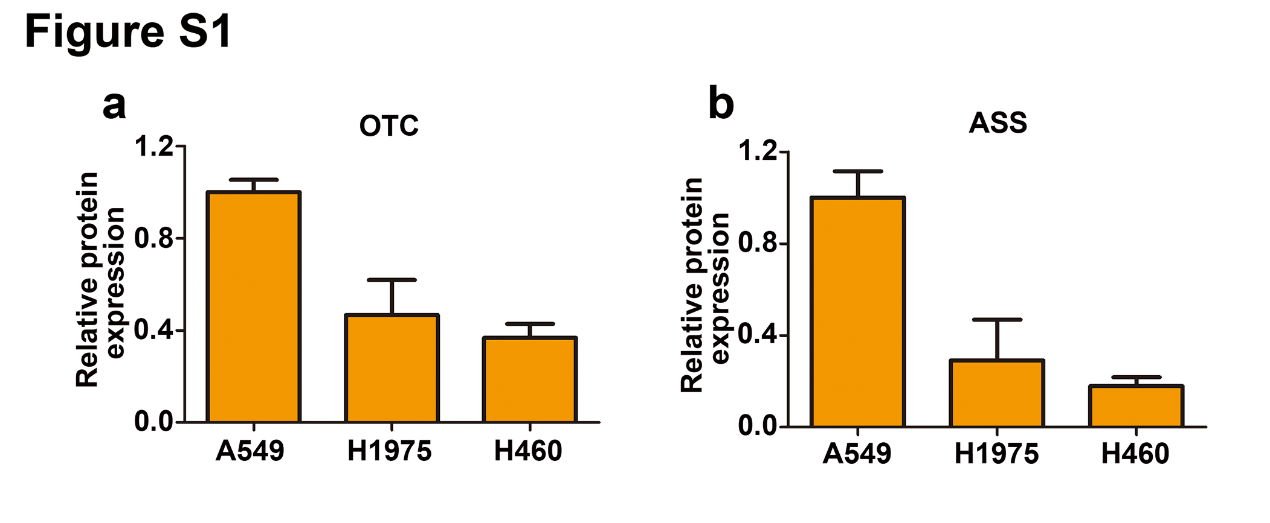


**Figure S2** **(a**-**f)** Statistics of the LC3-II, Cleaved-PARP and Cleaved-caspase 3 expression in Figure 5. Densitometric values were quantified using the ImageJ software and normalized to control. The values of control were set to 1. The data are presented as means ± SD of 3 independent experiments.


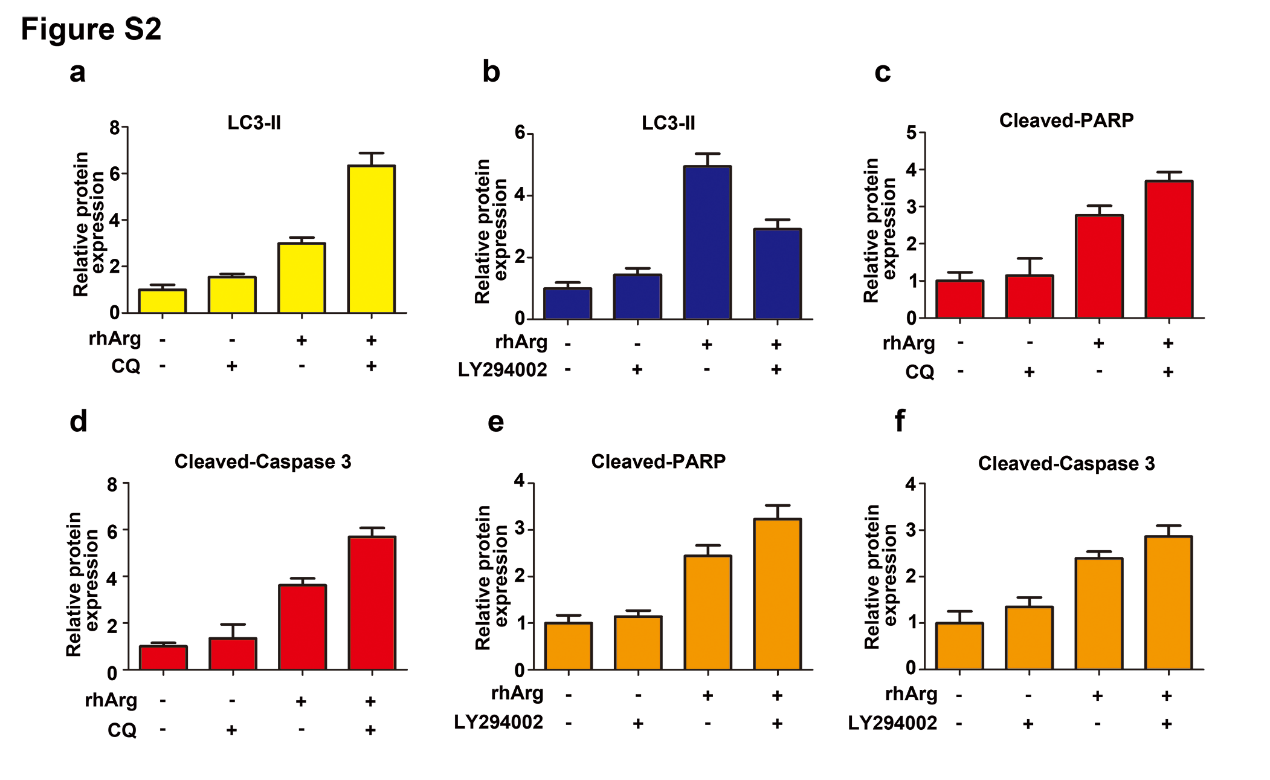


**Figure S3 (a** and **b)** Statistics of the Cleaved-PARP and Cleaved-caspase 3 expression in Figure 7D. Densitometric values were quantified using the ImageJ software and normalized to control. The values of control were set to 1. The data are presented as means ± SD of 3 independent experiments.


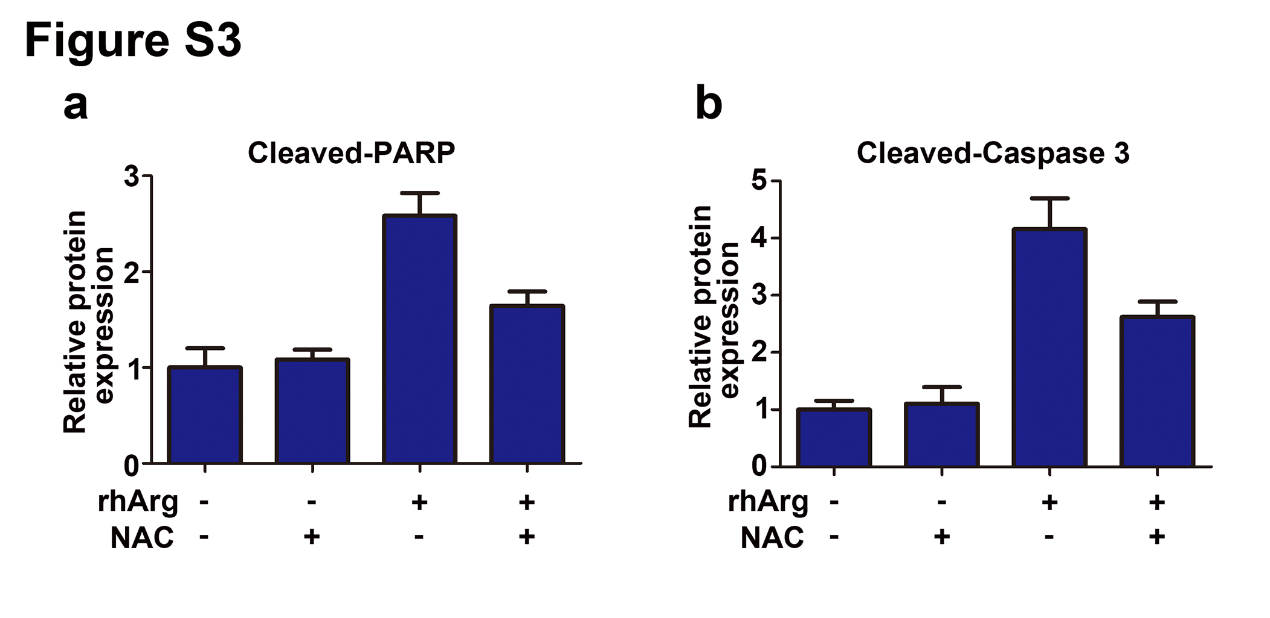


**Figure S4** Body weight of different groups was measured twice a week.


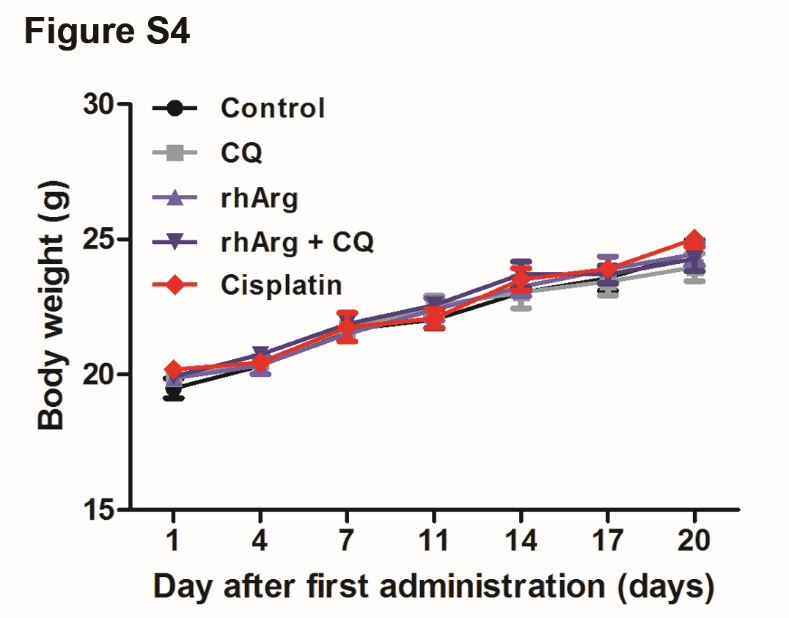

Supplement: Supplementary Information [file cddis2017137x1.docx]
